# Identification of Paralogous Life-Cycle Stage Specific Cytoskeletal Proteins in the Parasite *Trypanosoma brucei*

**DOI:** 10.1371/journal.pone.0106777

**Published:** 2014-09-02

**Authors:** Neil Portman, Keith Gull

**Affiliations:** 1 Sir William Dunn School of Pathology, University of Oxford, Oxford, United Kingdom; 2 Faculty of Veterinary Science, University of Sydney, Sydney, Australia; University of Texas Medical School at Houston, United States of America

## Abstract

The life cycle of the African trypanosome *Trypanosoma brucei*, is characterised by a transition between insect and mammalian hosts representing very different environments that present the parasite with very different challenges. These challenges are met by the expression of life-cycle stage-specific cohorts of proteins, which function in systems such as metabolism and immune evasion. These life-cycle transitions are also accompanied by morphological rearrangements orchestrated by microtubule dynamics and associated proteins of the subpellicular microtubule array. Here we employed a gel-based comparative proteomic technique, Difference Gel Electrophoresis, to identify cytoskeletal proteins that are expressed differentially in mammalian infective and insect form trypanosomes. From this analysis we identified a pair of novel, paralogous proteins, one of which is expressed in the procyclic form and the other in the bloodstream form. We show that these proteins, CAP51 and CAP51V, localise to the subpellicular corset of microtubules and are essential for correct organisation of the cytoskeleton and successful cytokinesis in their respective life cycle stages. We demonstrate for the first time redundancy of function between life-cycle stage specific paralogous sets in the cytoskeleton and reveal modification of cytoskeletal components *in situ* prior to their removal during differentiation from the bloodstream form to the insect form. These specific results emphasise a more generic concept that the trypanosome genome encodes a cohort of cytoskeletal components that are present in at least two forms with life-cycle stage-specific expression.

## Introduction

The African trypanosome, *Trypanosoma brucei* is a single-celled obligate parasite that causes African sleeping sickness. It has a complex lifecycle with multiple distinct phases encompassing a passage through an insect vector - the Tsetse fly - and a mammalian host [1]. From the midgut of the insect vector, parasites migrate to and colonise the salivary glands. When the fly takes a bloodmeal, parasites with pre-adaptations to the mammalian bloodstream are transferred to the mammalian host. An extracellular bloodstream infection is established in the host which eventually results in the production of parasites competent for transfer back to a Tsetse fly. This life-cycle involves both proliferative and non-proliferative stages, each with distinct morphology that reflect the very different environments encountered by the parasite [1]. The most widely studied life cycle stages are the procyclic form from the Tsetse midgut and the long slender form that colonises the mammalian bloodstream.

The various lifecycle morphologies range from extremely long, slender cells to relatively short, broad cells and life-cycle transitions are, in many cases, accompanied by rearrangements of the relative position of nuclei and kinetoplast (the concatenated mitochondrial DNA). Such drastic changes in morphology are accomplished through asymmetric cytokinesis [2] or through differentiation of growth arrested cells [3] but all must be accomplished within the continuous presence of the persistent microtubule cytoskeleton – a highly ordered array of subpellicular microtubules that underlies the plasma membrane.

The transition from the long slender bloodstream form to the procyclic form occurs via a growth arrested short stumpy bloodstream form [4]. This differentiation involves a range of metabolic and morphological changes including mitochondrial elaboration and exchange of the protective surface glycoprotein coat used in immune evasion. Although both cell types are morphologically trypomastigotes (i.e. the kinetoplast is positioned to the posterior of the nucleus and the flagellum is attached to the cell body for much of its length) bloodstream and procyclic forms differ in terms of the relative positions of the kinetoplast and the organisation of the nuclei and kinetoplasts during the cell cycle [1], [5]–[9]. The kinetoplast is positioned much closer to the posterior pole of the cell in the bloodstream form and in dividing cells the nuclei and kinetoplasts are arranged in a posterior-K-K-N-N-anterior morphology as opposed to the posterior-K-N-K-N-anterior morphology adopted by procyclic form dividing cells.

Notwithstanding the differences in morphology between bloodstream and procyclic forms, at the ultrastructural level the organisation and appearance of the microtubules of the subpellicular corset are indistinguishable. However, a growing number of cytoskeletal components have been identified that have undergone gene duplication with the resultant paralogous genes showing life-cycle stage-specific expression in *T. brucei* [10]–[15]. These include components of the subpellicular corset such as CAP5.5 and CAP5.5V as well as proteins involved in the Flagellum Attachment Zone (FAZ), a specialised domain of the cytoskeleton that connects the flagellum to the cell body.

Recent studies have suggested that the *T. brucei* lifecycle is accompanied by changes to the cell proteome [16], [17], but the extent and significance of the changes for certain cytoskeletal components remains unclear. We have performed a comparative proteomic analysis of isolated cytoskeletons from bloodstream and procyclic form cultures. We identified a novel pair of paralogous cytoskeletal proteins that show life-cycle stage specificity and have roles in the organisation of microtubules in the subpellicular corset in their respective life-cycle stages. We show that these two proteins exhibit redundancy of function when expressed in the exogenous life-cycle stage; the first demonstration of this phenomenon for *T. brucei* stage specific cytoskeletal components. Finally we provide evidence of protein modification prior to removal of one of these proteins during differentiation from the bloodstream form to the procyclic form.

## Results

### Candidate cytoskeletal proteins identified using comparative proteomics

**Figure 1:**
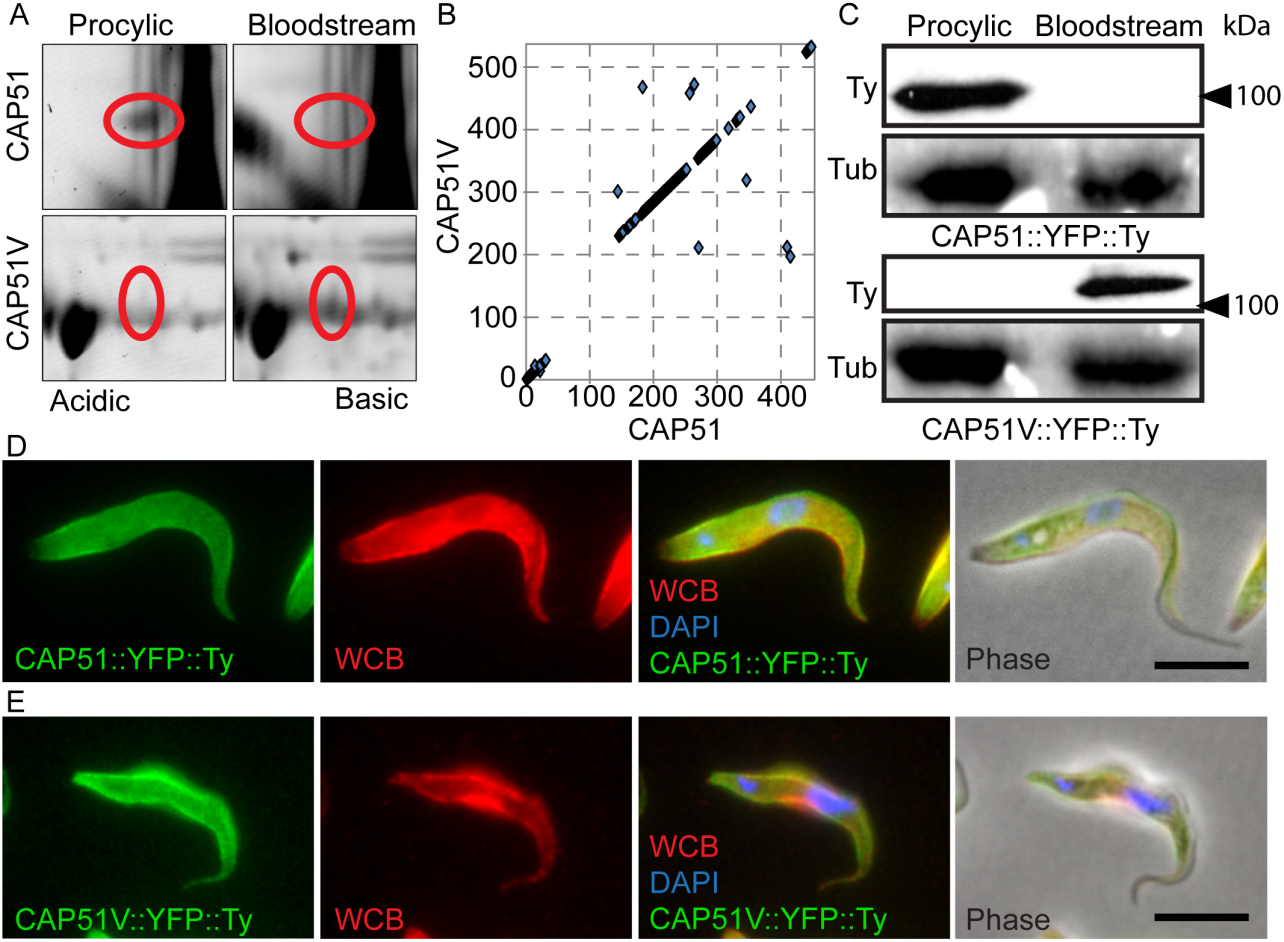
CAP51 and CAP51V are paralogous lifecycle stage-regulated cytoskeletal components. (A) Sections from a 2D-DiGE comparison of detergent extracted Bloodstream forms and Procyclic forms (Full gels in Figure S1) showing views of the gel regions corresponding to CAP51 and CAP51V. The spot corresponding to CAP51 is present in samples from procyclic form only and the spot corresponding to CAP51V has greater density in bloodstream form samples. (B) Dotplot alignment of CAP51 (x axis) and CAP51V (y axis) protein sequences using a 4 residue window. CAP51V contains a 75 residue lysine rich insert that is not present in CAP51. (C) Western blot of detergent extracted bloodstream and procyclic form cells expressing YFP::Ty tagged CAP51V or CAP51. CAP51V::YFP::Ty is detected only in the bloodstream form and CAP51::YFP::Ty only in the procyclic form. A ponceau stain of the membrane showing the tubulin (Tub) region is included as an indication of relative loading. (D) Procyclic form cells expressing CAP51::YFP::Ty. CAP51 localises to the whole of the subpellicular corset apart from the extreme posterior end of the cell and is not present on the flagellum. (E) Bloodstream form cells expressing CAP51V::YFP::Ty. CAP51V also localises to the subpellicular corset with no signal detected on the flagellum. Red = whole cell body (WCB), blue = DAPI, bar = 5 µm.

We compared the protein composition of detergent extracted bloodstream form cells to that of detergent extracted procyclic form cells using Difference Gel Electrophoresis (DiGE) as previously described [18] (Figure 1A, Figure S1). A total of 36 spots showing a difference in density between the two samples were cut from the gel for mass spectrometric analysis. This yielded 71 identifications based on at least two unique peptides and with a confidence interval greater than 99%, forming a non-redundant candidate set of 49 proteins. Both CAP5.5 and CAP5.5V were present in this set and showed greater abundance in samples from procyclic forms and bloodstream forms respectively. A total of 18 proteins were identified that are annotated as hypothetical at TriTrypDb.

Two of the hypothetical proteins identified - Tb927.7.2640 and Tb927.7.2650 - are encoded by open reading frames that are adjacent to one another on chromosome 7 and that are related but not identical.

Gel spots corresponding to Tb927.7.2640 appeared most strongly in procyclic form samples and spots corresponding to Tb927.7.2650 showed a greater density in bloodstream form samples. Tb927.7.2640 (procyclic form) has an apparent molecular weight of 51 kDa and hence we have named these proteins CAP51 (Tb927.7.2640) and CAP51V (Tb927.7.2650) in accordance with previous nomenclatures [11], [12].

Two-way alignment of the protein sequences showed high similarity for most of the length but with a region of very low similarity towards the N terminus (Figure 1B). This region is around 120 residues long in CAP51 and 195 residues long in CAP51V and in the latter case includes a complex lysine rich repetitive element. The 5′UTRs of the two genes are identical for around 200 bp upstream of the open reading frames but no significant similarity could be found between the 3′UTRs. Alignment to the NCBI non-redundant protein database by BLASTP showed that homologues to these proteins are restricted to kinetoplastids, with both *Leishmania major* (LmjF.22.0730) and *Trypanosoma cruzi* (Tc00.1047053506859.170) genomes encoding a single homologue. Querying either CAP51 or CAP51V protein sequences against the Pfam database revealed no known domains or motifs. Both proteins are predicted to form coiled-coils in the C-terminal conserved regions (via COILS, http://embnet.vital-it.ch/software/COILS_form.html).

### CAP51 proteins localise to the subpellicular microtubule corset

We introduced a C terminal YFP::Ty tag into one of the endogenous alleles for both proteins individually in both bloodstream and procyclic form cells, preserving the endogenous 3′UTRs of the genes and any regulatory signals contained therein. Western blot analysis of whole cell extracts from each of these cell lines using the anti-Ty tag monoclonal antibody BB2 (Figure 1C) confirmed that CAP51 is expressed in procyclic forms but is not expressed above the level of detection in bloodstream forms. CAP51V is expressed in bloodstream forms but is not detected in procyclic forms. Analysis of native YFP fluorescence in whole cells and detergent extracted cells with co-staining for the marker protein WCB [19] showed that both proteins localise to the subpellicular corset in their respective life cycle stages (Figure 1D, E). The YFP signal in both lifecycle stages decorated the entire subpellicular corset evenly, with the exception of the extreme posterior end of procyclic form cells, and persisted throughout the cell cycle. No YFP signal was detected in the flagellum in either life cycle stage.

### CAPs during differentiation

To examine the expression of CAP proteins during differentiation, we incubated the CAP51V::YFP::Ty and CAP51::YFP::Ty monomorphic bloodstream form cell lines at 28°C in the presence of 6 mM cis-aconitate, a treatment that has been shown to induce differentiation to procyclic forms in culture conditions [20].

**Figure 2:**
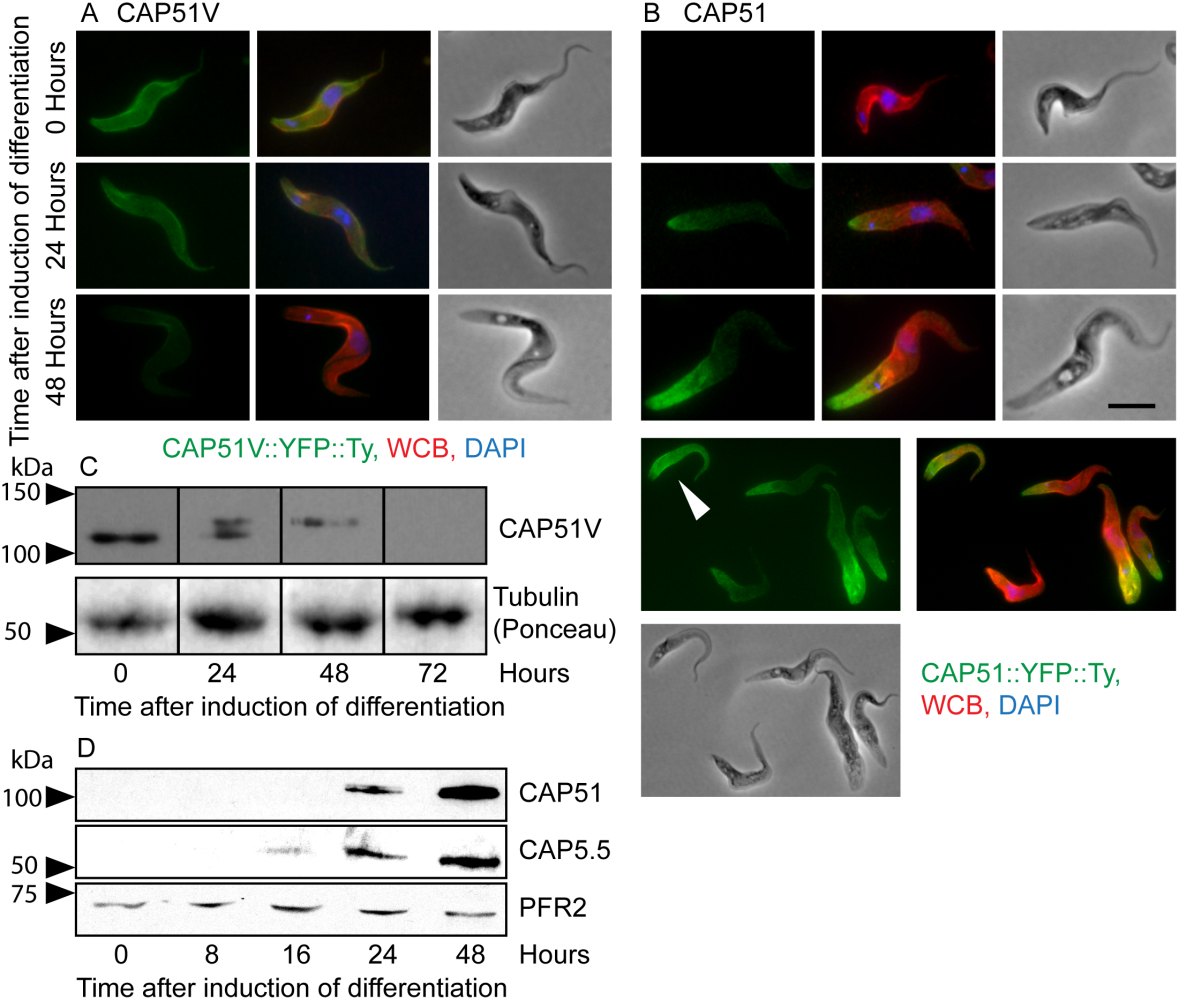
CAP proteins during differentiation Monomorphic bloodstream form cells with CAP51V::YFP::Ty or CAP51::YFP::Ty expressed from an endogenous locus were induced to differentiate into procyclic forms by the addition of cis-aconitate and incubation at 28°c. (A) CAP51V signal (green) disappears uniformly across the cell body during differentiation. (B) CAP51 signal (green) intrudes from the posterior end of the cell during the course of the differentiation and (C) cells with uniform fluorescence are detected by 48 hours after induction (white arrowhead). Red = WCB, blue = DAPI, bar = 5 µm. (D) Western blot of CAP51V::YTFP::Ty on detergent extracted cells using BB2. A single band is visible in bloodstream forms but over the time course a second band approximately 10 kDa larger than the first appears. Neither form of CAP51V is detectable by 72 hours after induction. Tubulin (ponceau) is shown as an indication of relative loading. (E) Western blot of CAP51::YFP::Ty on detergent extracted cells using BB2. CAP51 is undetectable in bloodstream form cells. A band of the correct predicted molecular mass appears by 24 hours, slightly later than the first detection of CAP5.5 (detected with the CAP5.5 antibody) at 16 hours. PFR2 (L8C4) is shown as an indication of relative loading.

CAP51V::YFP::Ty showed uniform fluorescence across the microtubule corset - as described above - which persisted through 24 and 48 hours after exposure to cis-aconitate but at a steadily decreasing intensity (Figure 2A, all images captured and processed using identical parameters). Western blot analysis of detergent extracted cells with BB2 (Figure 2D) showed that CAP51V::YFP::Ty was present as a single band of the correct apparent molecular weight in undifferentiating bloodstream forms. Surprisingly, by 24 hours after induction a second, larger band appeared and by 48 hours only the higher molecular weight band was detectable. By 72 hours no CAP51V::YFP::Ty was detectable. The apparent difference in molecular weight between the two CAP51V bands was approximately 10 kDa.

As before, CAP51::YFP::Ty was not detected before exposure to differentiation conditions. However, by 24 hours after induction of differentiation, fluorescence at the posterior end of cells was observed (Figure 2B). By 48 hours after induction of differentiation, cells with uniform fluorescence over the whole cytoskeleton with the exception of the posterior end, comparable to that seen in cultured procyclic forms, were observed (Figure 2C). CAP51::YFP::Ty was undetectable by Western blot analysis using BB2 at early time points but appeared between 16 and 24 hours after differentiation was induced (Figure 2E). This was slightly later than the appearance of CAP5.5 that was first detected 16 hours after induction of differentiation.

### Ablation of CAP proteins leads to aberrant morphology and compromised cytokinesis

**Figure 3:**
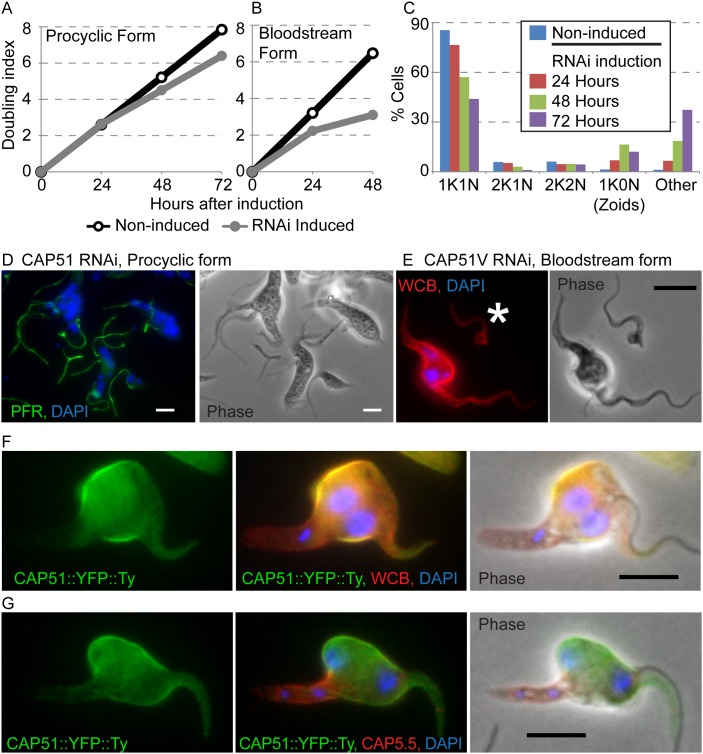
Ablation of CAP51 and CAP51V leads to reduced growth rate and aberrant morphology. (A, B) RNAi mediated ablation of CAP51 in procyclic forms (A) and CAP51V in bloodstream forms (B) results in reduced growth rate. Representative plots from three replicates are shown. Grey, RNAi induced; black non-induced. (C) Ablation of CAP51 in procyclic forms results in an accumulation of cells with aberrant nucleus/kinetoplast numbers including the production of 1K0N zoids. Cells with multiple nuclei (i.e. greater than 2) are classified as “Other”. Representative counts from three replicates are shown. (D) 72 hours after induction of RNAi against CAP51 in procyclic forms, multinucleate cells with multiple kinetoplasts and flagella and 1K0N zoids can readily be observed, green = PFR (L8C4). (E) RNAi against CAP51V in the bloodstream form. Cells undergo aberrant cytokinesis and cytoplasts that contain no nuclear or kinetoplast DNA (white asterisk) are also observed. Red = WCB. (F, G) RNAi against CAP51 in the procyclic form. CAP51 (green) is lost preferentially in the posterior portion of the cell which remains positive for other markers of the subpellicular corset (F) WCB (red) and (G) CAP5.5 (red). Cell morphology is distorted with the diameter of the midpoint of the cell being abnormally large compared to the anterior and posterior. (D–G) Blue = DAPI, bar = 5 µm.

We generated cell lines in both bloodstream and procyclic form cells with doxycycline inducible RNAi against each CAP mRNA individually, targeting the region of low similarity near the 5′ end of each coding sequence. Population doubling times were determined in both life-cycle stages in the presence and absence of RNAi against each protein. RNAi against either CAP resulted in a decrease in population growth rate compared to non-induced cells in the corresponding life cycle stage (Figure 3A, B). Cells with abnormal numbers of nuclei and kinetoplasts, including monstrous multinucleate cells (greater than 2N) and anucleate zoids, began to accumulate by 72 hours after induction of RNAi against CAP51 in procyclic forms (Figure 3C&D). In bloodstream forms, cells undergoing aberrant cytokinesis (Figure 3E) were evident by 24 hours after induction of RNAi against CAP51V and cytoplasts with no detectable DNA content appeared. In the reciprocal experiments, RNAi against CAP51V in the procyclic form or CAP51 in the bloodstream form had no effect on population growth rates or cell morphologies (not shown). Addition of a YFP::Ty tag to CAP51 in the CAP51 RNAi background showed that by 48 hours after RNAi induction, protein was lost preferentially in the posterior portion of the cell (Figure 3F, G). Loss of CAP protein did not affect the distribution of the cytoskeletal markers CAP5.5 in procyclic form or WCB in either life cycle stage. CAP51 ablation in procyclic forms resulted in cells exhibiting distorted morphology such that the diameter of the cell at the midpoint was abnormally large by 48 hours after induction compared to the rest of the cell body.

**Figure 4:**
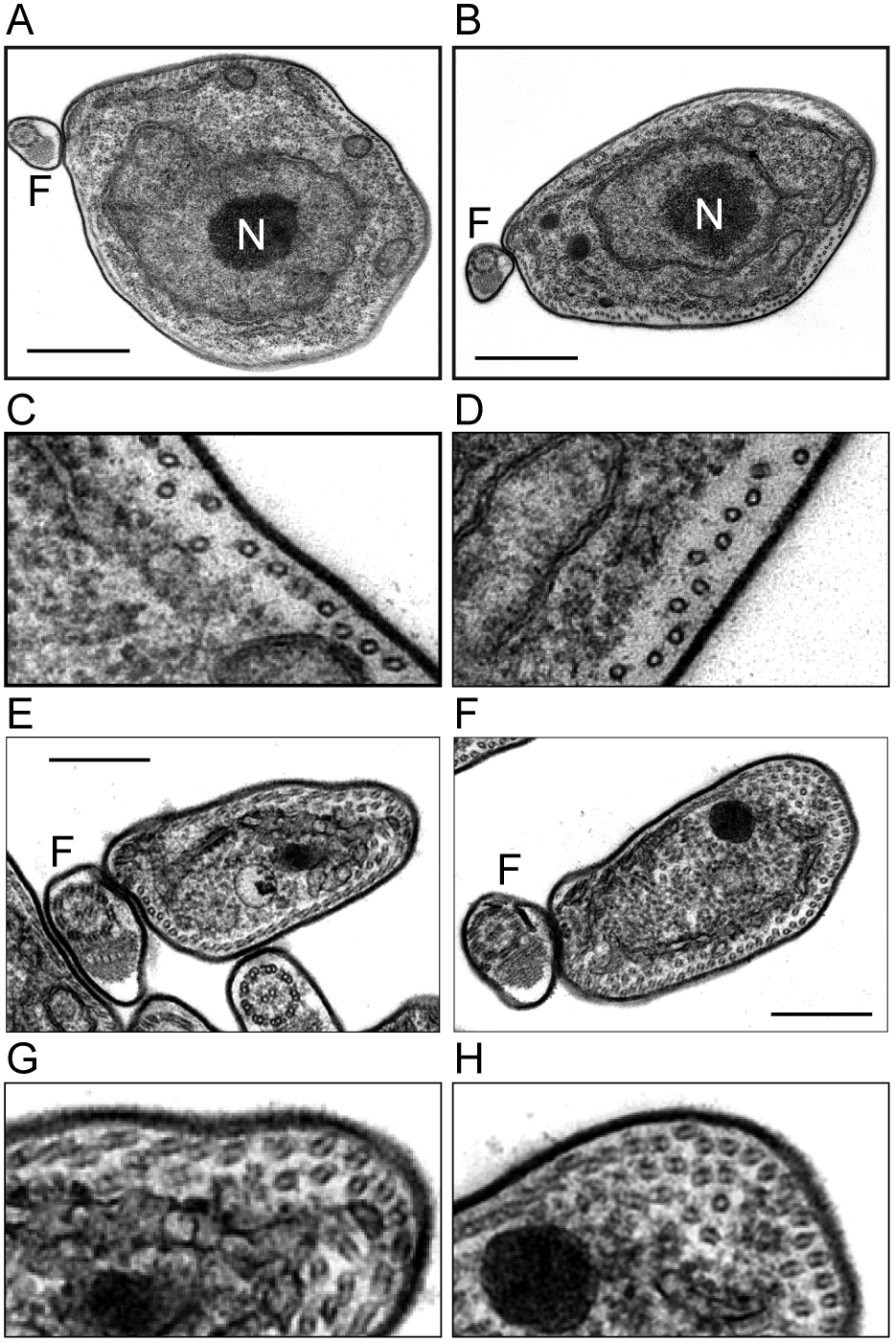
Ablation of CAP proteins disrupts the organisation of the microtubule corset. Thin section TEM of transverse sections through bloodstream form trypanosomes 24 hours after induction of RNAi against CAP51V. (A, B) The microtubules of the subpellicular array become disordered at the midpoint of the cell. Bar 800 nm (C, D) higher magnification view of part of the subpellicular array of A and B respectively. (E, F) Multiple layers of microtubules are present at the anterior end of the cell. Bar 400 nm (G, H) higher magnification view of part of the subpellicular array of E and F respectively. Similar disruptions to the organisation of the subpellicular corset can be seen in procyclic form sections 48 hours after induction of RNAi (not shown). N = Nucleus, F = flagellum.

Examination of bloodstream form cells 24 hours after induction of RNAi against CAP51V by thin section electron microscopy showed that the tight organisation of the subpellicular array of microtubules was lost as a result of RNAi (Figure 4). In wild-type cells the subpellicular microtubules form a regularly spaced single layer array closely apposed to the plasma membrane. In CAP51V-depleted bloodstream form cells, sections that contained a nucleus (i.e. around the mid-point of the cell, n = 72) showed disruptions to the subpellicular array with individual microtubules displaced along an axis orthogonal to the plane of the array (Figure 4, A–D). These perturbations in the microtubule array occurred in short runs with other areas of the array in the same cross-section appearing normal. In sections with an associated flagellar profile but no nucleus (i.e. an anterior position in the cell, n = 56), the disorganisation of the microtubule array was more pronounced with multiple layered sheets of microtubules apparent (Figure 4, E–H). Disorganisation of the microtubule array was observed rarely in cross sections without an associated flagellar profile (i.e. posterior to the flagellar pocket, n = 76).

### CAP functions are complementary

**Figure 5:**
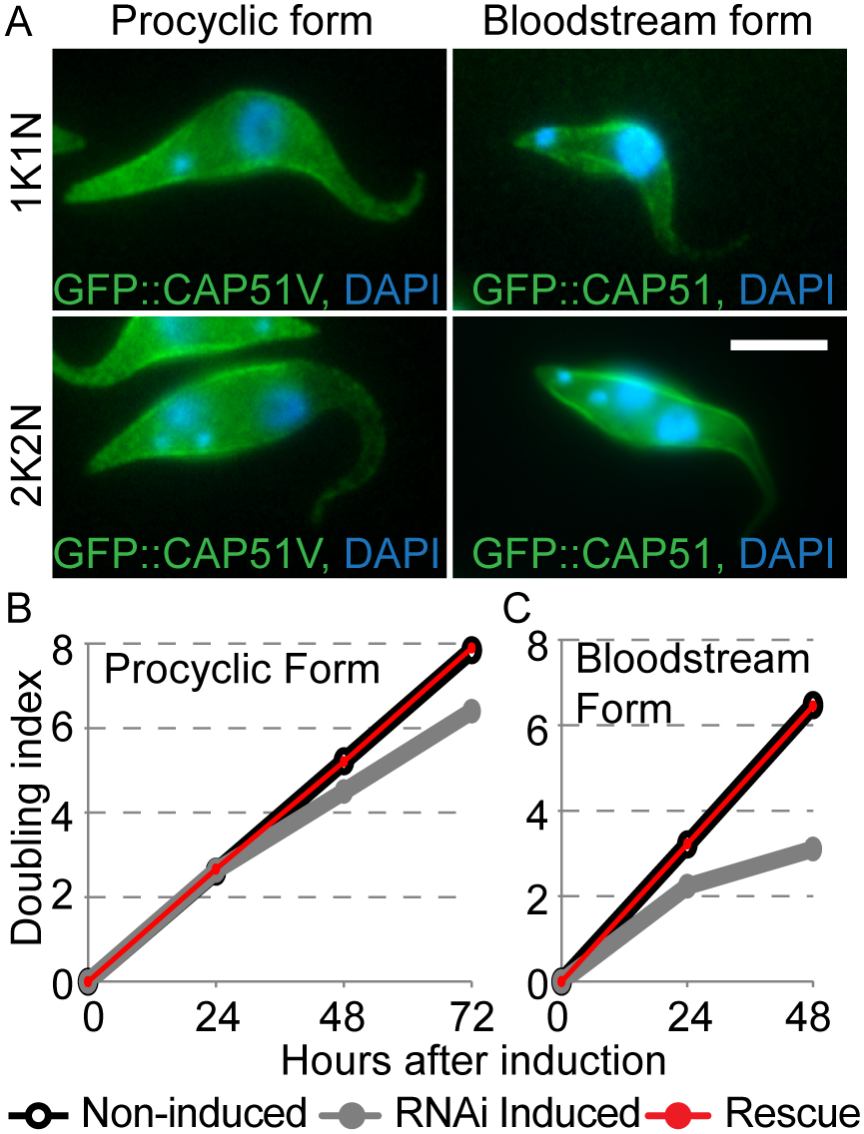
Localisation of CAP proteins to the subpellicular microtubule array is not life-cycle stage dependant. A) Ty::GFP::CAP51V and Ty::GFP::CAP51 expressed from inducible ectopic loci for 24 hours localise to the subpellicular array of procyclic form and bloodstream form cells respectively. Expression of the ectopic protein does not disrupt positioning of the nuclei and kinetoplasts of 2K2N cells in either life-cycle stage. Bar = 5 µm. (C, D) Simultaneous induction of RNAi against endogenous protein and expression of ectopic protein rescues the parental growth defect. (C) Ablation of CAP51 in procyclic forms reduces growth rate (grey) that is rescued by simultaneous expression of CAP51V (red). (D) Ablation of CAP51V in the bloodstream form reduces growth rate (grey) that can be rescued by simultaneous expression of CAP51 (red). Representative plots from three independent replicates are shown. Growth rates of non-induced cells (black) are comparable to that of rescued cells and expression of the endogenous protein with an N terminal GFP tag from an inducible ectopic locus did not rescue the growth defect (not shown).

Phenotypes associated with RNAi ablation of CAP proteins were similar in the two life-cycle stages suggesting that the two proteins have similar functions. We expressed Ty::GFP::CAP51V in procyclic forms and Ty::GFP::CAP51 in bloodstream forms from an inducible promoter. In both cases cells grew normally after induction and the ectopic protein localised to the subpellicular array (Figure 5A). When RNAi of the endogenous protein and ectopic expression of the exogenous protein were induced simultaneously, the growth rate/cell division defects were rescued in both life cycle stages (Figure 5B, C) and the subpellicular array appeared normal by TEM analysis (not shown). Simultaneous induction of RNAi against the endogenous protein and ectopic expression of the RNAi target protein failed to rescue the phenotype in either life-cycle stage (not shown).

## Discussion

### Paralogous cytoskeletal proteins with life-cycle stage-specificity

A growing number of cytoskeleton-associated proteins have been identified that show life-cycle stage-specific expression in *T. brucei*. Expression of CAP5.5 [10] and the related CAP5.5V [11] is restricted to the procyclic form and the bloodstream form respectively. Ablation of either protein in its endogenous life-cycle stage resulted in the accumulation of cells with abnormal numbers of nuclei and/or kinetoplasts, particularly the 1K0N cytoplasts known as zoids. The similarity in phenotypes suggests that CAP5.5 and CAP5.5V play analogous roles in their respective life-cycle stages. Another pair of related cytoskeleton associated proteins, CAP15 and CAP17, have been shown to stabilise microtubules and exhibit regulated expression in the life cycle [12]. Both proteins have analogous localisations at the anterior end of the subpellicular corset and overexpression of either in procyclic forms resulted in a similar organelle positioning/cytokinesis defect phenotype.

There is now evidence that several components of the FAZ are present as paralogues in the genome and exhibit life-cycle regulated expression profiles. FLA1, an essential component of the FAZ, is significantly up-regulated in the procyclic form [21] whereas the recently identified FLA2, which is highly similar to FLA1 but contains a 44 residue proline rich insert [13], is significantly up-regulated in the bloodstream form. The transmembrane protein FLA3 also has a crucial role in flagellum attachment but only appears to be expressed in the bloodstream form [14]. A so-far unnamed protein, related to FLA3 and with similar domain architecture appears to be significantly upregulated in procyclic forms [14]. Our own data support both procyclic form specific expression and FAZ localisation for this protein [15]. Similarly as with FLA1/FLA2, a distinguishing feature of this pair is a 30 amino acid insert in the bloodstream form protein.

Using DiGE we identified numerous proteins that showed greater abundance in one or other of the two lifecycle stages analysed. These included both CAP5.5 and CAP5.5V that showed greater abundance in samples from the appropriate life-cycle stage (procyclic form and bloodstream form respectively [11]), hence validating our approach. Two pairs of hypothetical proteins in the dataset shared an orthologue group and of these CAP51 and CAP51V exhibited a pattern of life-cycle stage regulated expression. This pattern is supported by data from two recent global comparisons of protein expression in the two life-cycle stages [16], [17]. The genomes of *L. major* and *T. cruzi* both encode a single CAP orthologue and BLASTP analysis detected no related proteins outside the *Kinetoplastida*.

Both proteins localise to the subpellicular microtubule array evenly throughout the cell cycle, with the exception of the extreme posterior end of procyclic form cells where no CAP51 was detected. Several studies have shown that there are differences in the regulation of microtubules at the posterior end of procyclic and bloodstream form cells, strikingly displayed by the nozzle phenotype in the procyclic form [22]–[24]. We speculate that this specific regulation of microtubule extension in procyclic forms requires the exclusion of certain cytoskeletal components from the posterior end of the cell.

### CAP51V is modified during differentiation

During differentiation of monomorphic bloodstream form cells to procyclic form, CAP51 first appeared at the posterior end of cells between 16 and 24 hours after induction of differentiation and spread towards the anterior end of the cell to give a uniform distribution similar to that seen in cultured procyclic forms - a pattern consistent with that observed previously for CAP5.5 [25]. By contrast, CAP51V disappeared evenly across the whole cell body during differentiation. Analysis of the cells by Western blot showed that this was accompanied by an apparent increase in the molecular weight of a proportion of the protein by 24 hours with all remaining protein present at the higher molecular weight by 48 hours. This shift in mobility on the gel corresponds to an increase in molecular weight of approximately 10 kDa which is consistent with the approximate shift that would result from the addition of ubiquitin. Ubiquitination is associated with marking proteins for degradation or for relocation in the cell and this would certainly correlate with the observed behaviour of CAP51V during differentiation. Interestingly, the samples used for Western blot analysis were treated with detergent prior to electrophoresis of the insoluble fraction so the apparent modification of CAP51V must take place whilst it is still associated with the cytoskeleton.

### CAP proteins function in the organisation of subpellicular microtubules

RNAi-mediated knockdown of both CAP proteins in their native life-cycle stages resulted in the accumulation of cells with aberrant morphologies. CAP proteins were lost first from the posterior end of cells after RNAi induction. During the cell cycle, cells become both longer and wider prior to cell division [9], [26], [27]. Length is increased by the extension of the subpellicular microtubules into the posterior end of the cell. With the exception of a specialised quartet of microtubules associated with the FAZ, all of the subpellicular microtubules are orientated with their more dynamic plus ends towards the posterior of the cell [28]. Width is increased by the intercalation of very short microtubules into the array which then extend between the existing microtubules [5], [9]. In order for this to occur the existing intermicrotubule connections must be severed. These mechanisms may go some way to explain the pattern of loss of CAP proteins - within a single cell cycle, microtubules at the posterior of the cell extend in the absence of new protein. The apparent loss of protein at the posterior of the cell would therefore more properly be considered a loss of gain of protein. Although the loss of gain would also presumably be occurring at the sites (or potential sites) of new microtubule intercalation [9], [24], [29], this may be disguised at the level of immunofluorescence analysis by the presence of old protein associated with the existing microtubule array. An opposing model would be that during the extensive remodelling associated with cell growth and division, cytoskeletal proteins are actively stripped from microtubules during microtubule elongation and intercalation and rapidly replaced. In this way an absence of new protein would be most readily observed at the dynamic posterior end of the cell. This pattern of protein loss is consistent with that previously described for RNAi against other cytoskeletal proteins such as CAP5.5 and WCB [11], [19].

At time points prior to the appearance of large numbers of multi-nucleate cells, 2N cells exhibited a particular morphology where the diameter of the mid-portion of the cell appeared abnormally large. Our analysis of mutant cells by TEM showed that this morphology is accompanied by disruptions to the organisation of the subpellicular microtubule array, with a loss of the regular, ordered arrangement of microtubules into a continuous sheet seen in wild type cells. This loss of organisation is similar to that previously reported for RNAi against CAP5.5 and CAP5.5V [10], [11]. Here it was suggested that the phenotype observed may be due to defects in the process of either severing or establishing the inter-microtubule-connections necessary for the intercalation and extension of new microtubules into the sheet during the cell cycle [5]. We hypothesise that CAP51/51V also play a role in inter-microtubule connections. The increase in cell width in the midportion of cells depleted for CAP51 is consistent with a model whereby short nucleating microtubules are incorporated into the array as normal but further intercalation of the growing ends as they extend away from the point of insertion is prevented. Our TEM observations of disruptions to the microtubular array supports the view that microtubules are forced to extend outside the plane of the array but do seem to maintain some connection to the existing microtubules. The appearance of layered sheets of microtubules at the anterior ends of the cell can then be explained as microtubules extending in different planes being constricted into the smaller cell body diameter at the anterior end of the cell.

### Why does the cell require independently regulated versions of some cytoskeletal components?

Trypanosomes are unusual amongst eukaryotes in that genes are transcribed as polycistronic units [30]. In general genes do not contain introns and regulation of gene expression is accomplished post-transcriptionally. This means that all protein diversity must be encoded as discreet open reading frames (there is no opportunity for alternative splicing). We therefore hypothesised that two versions of CAP51 were present in the genome to address different functional requirements between life cycle stages. However, we found that expressing CAP51V in procyclic forms or CAP51 in bloodstream forms with simultaneous ablation of the endogenous protein completely rescued the RNAi phenotype. The ectopic protein localised to the subpellicular array and rescued cells exhibited normal morphologies. It is possible that over time small, initially undetectable aberrant phenotypes might accumulate although rescued cells have been kept in culture under induction for several weeks with no apparent detrimental effects. It is also possible that residual expression of the endogenous protein, bolstered by the presence of ectopic protein, is sufficient for normal cell functions. However, our data suggest that as well as being complementary when expressed ectopically, the proteins perform equivalent functions in their respective life-cycle stages. It appears that in terms of function within the cytoskeleton, these two proteins are essentially identical.

So why else might the cell require two independently regulated versions of the same protein? Differential CAP functions may be required under conditions to which the cells are not subjected in culture, or in life-cycle stages that were not examined in this study. Alternatively it may be that the regulation of expression itself that is important, i.e. it is the difference between the 3′UTRs that is critical. Being able to regulate each open reading frame individually may give the cell more fine control over gene expression than could be afforded by regulatory elements associated with a single open reading frame. Perhaps it is during the restructuring processes that occur in the life-cycle rather than those that occur in the individual cell cycles that such fine control becomes critical.

## Conclusion

The organisation and regulation of the cytoskeleton are key factors in the success of *T. brucei*, driving the morphological adaptations to each new environment encountered by the parasite through its life cycle. The extent to which the molecular composition of the cytoskeleton is life-cycle stage dependant is gradually being explored. There is now evidence for a number of stage-specific proteins, encompassing both the microtubule array and even more specialised cytoskeletal structures such as the FAZ. That many of these proteins exist as paralogous sets likely reflects the unusual arrangement of the trypanosome genome with its almost complete lack of introns and alternative splicing. However, the reasons for encoding similar proteins with similar functions for stage specific expression remain unclear. As our knowledge of these paralogous sets improves, our understanding of the regulation of the cytoskeleton through the life cycle will also increase.

## Materials and Methods

### Plasmid construction

Primers used in this study are presented in Table S1. RNAi constructs were generated by amplification of 300–700 bp of the target open reading frame from genomic DNA and insertion between the XbaI sites of p2T7-177 [31]. For ectopic expression of recombinant proteins with an N terminal Ty::GFP tag from a constitutive rDNA promoter, the entire open reading frame of the gene of interest was amplified and inserted in frame between the XbaI and BamHI restriction sites of pDex577-G [32]. For the expression of chimeric proteins with a C terminal YFP::Ty tag from endogenous loci, 150–250 bp of in-frame sequence up to but not including the stop codon and 150–250 bp of the 3′UTR of genes of interest were amplified and inserted in this order between the HindIII and SpeI restriction sites of pEnT6B-Ty::YFP::Ty, separated by an XhoI restriction site. The entire intergenic region downstream of the gene of interest was amplified and inserted between the BamHI and SphI restriction sites to preserve any regulatory elements in the 3′UTR.

### Trypanosome cell culture

Procyclic forms (427) were cultured at 28°C in SDM-79 medium supplemented with 10% v/v foetal bovine serum (Gibco) [33]. Bloodstream forms (927) were cultured at 37°C with 5% CO_2_ in HMI-9 medium supplemented with 15% v/v foetal bovine serum (Gibco). Cells were diluted as necessary to maintain the culture in log-phase. Cell density was measured using a Casy-counter (Model TT; Sharfe systems).

Induction of RNAi or ectopic gene expression was achieved by the addition of doxycycline to the culture medium to a final concentration of 1 µg ml^−1^.

Differentiation of monomorphic bloodstream forms to procyclic forms was induced by the addition of 6 mM cis-aconitate to the culture medium and transfer of the culture to 28°C.

### Transmission Electron Microscopy

For TEM cells were fixed in culture as previously described [34]. Following embedding and sectioning, samples were viewed in an FEI Tecnai-F12 electron microscope (FEI Company Ltd.) operating at 80 kV.

### Preparation of cells for light microscopy

Cells were harvested by centrifugation washed once in PBS and resuspended in PBS to a density of 1×10^7^ cells ml^−1^. Cells were then settled onto glass slides and washed in PBS.

For whole cells: cells were fixed with 3.7% w/v paraformaldehyde for 20 minutes before permeabilisation by incubation in methanol for 20 minutes at –20°C.

For cytoskeletons: cells were washed once with 1% v/v Nonidet P40 (Sigma) in PEME and fixed by incubation in methanol for 20 minutes at –20°C.

Immunoglobulin isotype-specific secondary antibodies conjugated to either fluorophore 488 or fluorophore 594 (Alexafluor, Invitrogen) were used to visualise antibodies. Samples were mounted in Vectashield mounting medium with 4′, 6′ - diamino-2-phenylindole (Vector Laboratories Inc) for visualisation of DNA and examined on a Leica DM5500B. Primary antibodies used in this study are as follows: BB2 – Ty epitope [35]; L8C4 – PFR2 [36]; WCB – Whole Cell Body [37]; CAP5.5 – CAP5.5 [10]. All image processing and analysis was performed in ImageJ (http://rsbweb.nih.gov/ij) using built in functions or custom scripts.

### Protein electrophoresis

Protein samples were prepared as previously described [34]. For Western blot analysis either 5×10^6^ (whole cell) or 1×10^7^ (cytoskeleton) cell equivalents were separated by SDS PAGE.

DiGE comparison of protein samples and mass spectrometric identification of peptides were conducted as previously described [18].

## Supporting Information

Figure S1 **2D-DiGE comparison of detergent extracted (A) Bloodstream forms and (B) Procyclic forms.** Spots that showed a twofold or greater change between the samples are circled. (C) and (D) show close up views of the gel regions corresponding to CAP51V and CAP51 respectively.
(TIF)

Table S1 **PCR Primers used in this study.**
(DOCX)
