# Supplementary material for: Identification of Paralogous Life-Cycle Stage Specific Cytoskeletal Proteins in the Parasite Trypanosoma brucei
Source: PLoS One. 2014 Sep 2;9(9):e106777. doi: 10.1371/journal.pone.0106777 (PMC4152294; doi:10.1371/journal.pone.0106777)
Supplement: Table S1 — PCR Primers used in this study. (DOCX) [file pone.0106777.s002.docx]

| CAP-51 RNAi | ORF RNAi target | ATATATTCTAGAAAGGATGAGGCGGAAAGTG |
| --- | --- | --- |
|  |  | ATATATTCTAGAAGCACCCCACATTATTGACG |
| CAP-51V RNAi | ORF RNAi target | ATATATTCTAGACATTAAGGAGTGACCGGAAA |
|  |  | ATATATTCTAGACTTCTTCCGCTTTTTGCTTT |
| CAP-51 OX | Ectopic expression | ATATATTCTAGAAGCACCACTGCACTGTCTTC |
|  |  | AGCGTAATAGGATCCTATTTGTCG |
| CAP-51V OX | Ectopic expression | ATATATTCTAGAAGCACCACTGCACTGTCTTC |
|  |  | ATATATGGATCCTCAGTAGTTGGCTTCACGAGC |
| CAP-51 Tag | ORF intergration | ATATATCTCGAGTTGAAGGGTTAAAGCGTTTGTT |
|  |  | ATATATACTAGTTTTGTCGGCCTCCCG |
|  | 3'UTR intergration | ATATATAAGCTTGATCGTATTACGCTCCATGTTCA |
|  |  | ATATATCTCGAGCGCCGTGGTTATCGTAAACT |
|  | 3'UTR full length | ATATATGCATGCTCGGCCTAAGAGATCGAATG |
| CAP-51V Tag | ORF intergration | ATATATCTCGAGAGCTGAAGGATGCGAAAGTC |
|  |  | ATATATACTAGTGTAGTTGGCTTCACGAGCAC |
|  | 3'UTR intergration | ATATATAAGCTTGGTACGCCCGCTCACAC |
|  |  | ATATATCTCGAGCTTCGAGTGCCTTCCTTGAC |
|  | 3'UTR full length | ATATATGCATGCAACGGAACCTTTTTCTTTTTCA |

Table S1. PCR primers used in this study
